# Nonionic surfactants and their effects on asymmetric reduction of 2-octanone with *Saccharomyces cerevisiae*

**DOI:** 10.1186/s13568-018-0640-1

**Published:** 2018-07-06

**Authors:** Yunquan Zheng, Liangbin Li, Xianai Shi, Zhijian Huang, Feng Li, Jianmin Yang, Yanghao Guo

**Affiliations:** 1 College of Chemistry, Fuzhou University, 2 Xueyuan Road, Fuzhou, 350116 China; 2 College of Biological Science and Engineering, Fuzhou University, 2 Xueyuan Road, Fuzhou, 350116 China; 3 Fujian Provincial Cancer Hospital, 420 Fuma Road, Fuzhou, 350014 China; 4 Fujian Key Laboratory of Medical Instrument and Pharmaceutical Technology, Fuzhou University, 2 Xueyuan Road, Fuzhou, 350116 China

**Keywords:** Nonionic surfactant, 2-Octanone, *Saccharomyces cerevisiae*, Asymmetric reduction

## Abstract

In an aqueous buffer system, serious reverse and side reactions were found in the asymmetric reduction of 2-octanone with *Saccharomyces cerevisiae*. However, some nonionic surfactants added to the aqueous buffer system improved the bioreduction process by decreasing the reverse and side reaction rates in addition to effectively increasing the average positive reaction rate. Further, a shorter carbon chain length of hydrophilic or hydrophobic moieties in surfactants resulted in a higher yield of (*S*)-2-octanol. The alkylphenol ethoxylate surfactants had a less influence than polyoxyethylenesorbitan trialiphatic surfactants on the product *e.e*. It suggested that the product *e.e.* resulting from the change of carbon chain length of the hydrophobic moieties varied markedly compared with the change of carbon chain length of the hydrophilic moiety. Emulsifier OP-10 and Tween 20 markedly enhanced the yield and product *e.e.* at the concentration of 0.4 mmol L^−1^ with a yield of 73.3 and 93.2%, and the product *e.e.* of 99.2 and 99.3%, respectively, at the reaction time of 96 h.

## Introduction

The yeast *Saccharomyces cerevisiae* contains alcohol dehydrogenases and other redox enzymes (Berlowska et al. 2006; Heidlas et al. 1991; Leskovac et al. 2002; Xu et al. 2005; De Smidt et al. 2008, 2012) which can catalyze bioreduction of ketones to optically active alcohols (Hummel 1997; Heidlas and Tressl 1990; Jung et al. 2010; Gonzalez et al. 2000). For example, the reduction performed by *S. cerevisiae* with 2-octanone yields (*S*)-2-octanol (Li et al. 2007; Dai and Xia 2006), an important intermediate for chiral synthesis (Threeprom 2007) and a model compound for studying keto bioreduction (Rundback et al. 2012). However, toxic hydrophobic substrates and/or products in the reaction result in a low yield and enantiomeric excess (*e.e.*) of products due to the reversible and side reactions caused by excessive substrates and/or products.

Supplementation of culture medium with surface-active agents shifts the physiologic properties of yeast and other cells (DeSousa et al. 2006; Aguedo et al. 2004; Vasileva-Tonkova et al. 2001; Laouar et al. 1996; Koley and Bard 2010). Factors affecting the status of biocatalysts, or the distribution or concentration of substrates and products in the cells, or altering the interaction between biocatalysts and substrates or products, impact the reaction rate and enantioselectivity. Therefore, surfactants are associated with these functions, (1) solubilization of hydrophobic substrates and products in the medium (Ganesh et al. 2006; Mahdi et al. 2011; Wang et al. 2004; Walters and Aitken 2001) to avoid their enrichment in the cell membranes, which reduce their concentration in cells; (2) formation of mixed micelles including surfactants and cell membranes (Hu et al. 2012; Liu et al. 2016; Kurakake et al. 2017), which affecting the structure and permeability of cell membrane, leading to altered physiology of cells and transmembrane transportation of substrates and products; (3) interaction of surfactants and intracellular enzymes, which changes the enzymatic characteristics and shifts enzymatic activities and enantioselectivities in asymmetric reactions (Awasthi et al. 2005; Roberts et al. 1977).

Therefore, bioreduction of toxic hydrophobic 2-octanone can be improved with appropriate surfactants in the medium. Until now, studies reported the influence of cloud-point system involving binding of nonionic surfactants with microbial cells and the effects of surfactants on microbial cell-mediated asymmetric reactions (Dominguez et al. 2003; Wei et al. 2003; Wang 2005; Goswami et al. 2000; Shi et al. 2010; Liu et al. 2017). However, limited research exists on the relationship between the properties of nonionic surfactants and their effects on asymmetric reduction of 2-octanone with *S. cerevisiae*.

## Methods and materials

### Strains and chemicals

*Saccharomyces cerevisiae* FD-12 was isolated from *S. cerevisiae* Type II, Sigma, USA.

2-Octanone was purchased from Merck-Schuchardt (Germany). (*R*)-2-octanol, (*S*)-2-octanol and (*S*)-(+)-PEIC (phenylethyl isocyanate) were supplied by Aldrich (USA). Emulsifier OP-6 (MW470), Tween 20 (MW1226.5), Tween 40 (MW1284.6), Tween 60 (MW1311.7) and Tween 80 (MW1309.6) were products of Sinopharm Chemical Reagent Co., Ltd. (China). Emulsifier OP-8 (MW559), Emulsifier OP-10 (MW647), sodium dodecyl sulfate (SDS), Emulsifier OP-12 (MW735) and cetyltriethylammonium bromide (CTAB) were obtained from Shanghai Sangon Biological Engineering Technology and Service Co., Ltd. (China). All other chemicals were of analytical grade. Glucose toolkit was purchased from BHKT Clinical Reagent Co. Ltd. (China).

### Activation of cells

Medium (g L^−1^): glucose 10.0, (NH_4_)_2_SO_4_ 5.0, KH_2_PO_4_ 1.0, MgSO_4_·7H_2_O 0.5, KCl 0.5, ZnSO_4_ 0.01, Fe_2_(SO_4_)_3_ 0.01, natural pH.

Conditions: 100 mL medium with 10 g lyophilized cells in a 250 mL-flask was placed in a rotary incubator set at 32 °C and 200 r min^−1^ for 2 h. The cells were harvested by centrifugation at 4000×*g* for 10 min and washed twice with Tris–HCl buffer solution (50 mmol L^−1^, pH8.0). The harvested cells were used for the following experiments.

### Analytical methods

The concentrations of 2-octanone and 2-octanol were determined by GC (Shimadzu GC-14C, Japan) equipped with a flame ionization detector and a non-polar fused silica capillary column AC1-0.25 (i.d. 0.25 mm, length 30 m, SGE, Australia). The GC conditions included N_2_ (99.999%) as the carrier gas, with a pressure front of the column at 100 kpa, the detector temperature 210 °C, the injector temperature 190 °C and the column temperature 170 °C.

The enantiomer resolution was based on the derivation of 2-octanol with optically pure isocyanate. 10 μL sample was mixed with 50 μL toluene and 2 μL (*S*)-(+)-PEIC and then left at 45 °C for 2 h. A fused silica capillary column and carrier gas (with column pressure front 65 kpa) were used. The injector and the detector were maintained at 250 and 270 °C, respectively. The retention times of (*R*)- and (*S*)-enantiomer were 18.8 min and 19.5 min.

Reaction rate (*v*) was calculated using the concentration of 2-octanol (*c*_*2*-*ol,t*_) as a function of time (t)

$$v = c_{2 - ol,t} / {\text{t}}$$

During the initial 3 h, the *v* denotes the initial reaction rate (*v*_0_). At a reaction time of 96 h, the *v* represents the average reaction rate.

$${\text{Yield }}\left( \% \right) = c_{2 - ol,t} /c_{2 - one,0} \times 100\%$$

*c*_*2*-*ol,t*_ is the concentration of 2-octanol (*c*_*2*-*ol,t*_) formed at time (t), and *c*_*2*-*one,0*_ is the concentration of 2-octanone before the reaction.

Enantiomeric excess of (*S*)-2-octanol

$$e.e. = \, \left( {{\text{A}}_{S} - {\text{ A}}_{R} } \right)/\left( {{\text{A}}_{S} + {\text{A}}_{R} } \right) \times 100\%$$

A_*S*_ and A_*R*_ are the peak areas of (*S*)-2-octanol and (*R*)-2-octanol, respectively.

### Viability assay

The viability of the cells was measured as the ratio of the living cell number after anaerobic pretreatment to the original living cell number. Pretreatment entailed mixing of 3.0 g wet cells with 20 mL Tris–HCl buffer, 0–2.0 mmol L^−1^ surfactants assayed and 0.2 g glucose at 32 °C and 200 r min^−1^ for 24 h. Using methylene blue, the living cells and the dead cells were distinguished and counted microscopically.

### Cell membrane permeability

Under conditions similar to viability assay and after culture up to 24 h, 2-mL broth was sampled and centrifuged at 10,000×*g* for 5 min. After appropriate dilution, the supernatant was used to determine the optical density at 260 and 280 nm, with a Shimadzu UV-1700 spectrophotometer using the broth cultured for 0 h as the control.

### Bioreduction assay

A normal reduction of 2-octanone in an aqueous solution was conducted in a 100-mL shake flask. 3.0 g wet cells were suspended in 20 mL Tris–HCl buffer with 0.2 g glucose. 2-Octanone was added to the medium to the final fixed concentration. The medium was placed in a rotary incubator at 32 °C and 200 rpm. At time intervals, 500 μL medium was withdrawn and extracted with 500 μL *n*-hexane for three times. The extracts were mixed for GC determination.

## Results

### Effect of surfactants on asymmetric reduction of 2-octanone

**Fig. 1:**
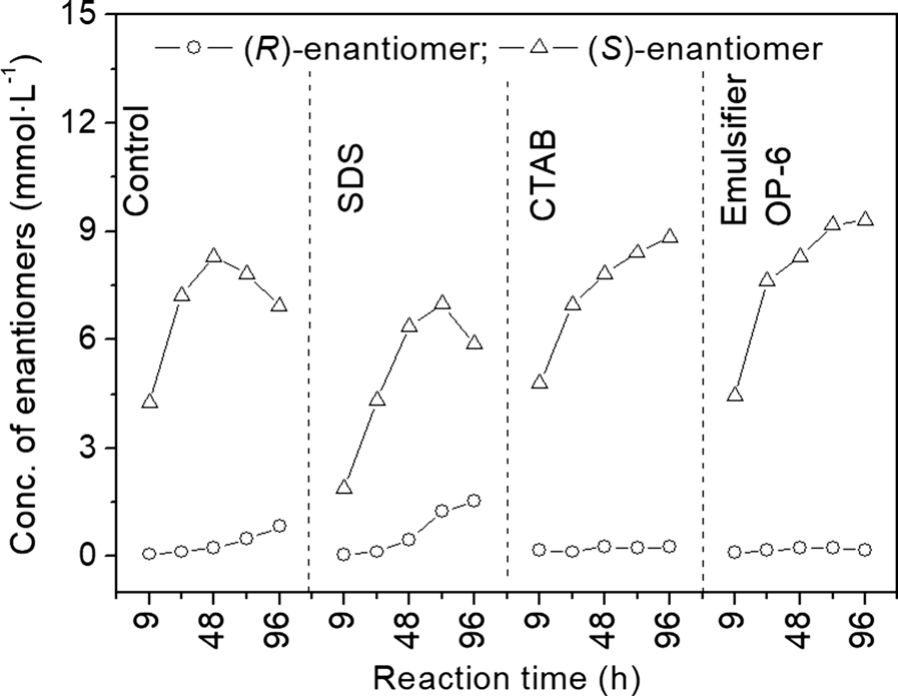
Time course of two enantiomers of 2-octanol in the reaction systems containing surfactants

As typical ionic surfactants, the anionic SDS (CH_3_(CH_2_)_11_SO_4_^−^) and cationic CTAB (C_16_H_33_N(CH_3_)_3_^+^), are different from the nonionic surfactant Emulsifier OP-6. Emulsifier OP-6 consists of alkylphenol ethoxylates (APEO) and does not dissociate in water. Nonionic surfactants show interesting features such as higher stability, lower surface tension, lower critical micelle concentration, higher aggregation number of micelles and better solubilization. To investigate the effect of surfactants in asymmetric reduction of 2-octanone with *S. cerevisiae*, we introduced surfactants into the aqueous media and the yield and product *e.e.* value of the reaction were analyzed. The results are shown in Fig. 1, the reduction of 2-octanone to (*S*)-2-octanol catalyzed by the whole cell *S. cerevisiae*, led to low yield and product *e.e.* value. Accordingly, the presence of Emulsifier OP-6 and CTAB, except SDS, showed a better yield and product *e.e.* value. The nonionic Emulsifier OP-6 was the best of the surfactants investigated.

In the reaction system with surfactants, it was apparent that not only the positive reaction was improved, but also the side and reverse reactions inhibited by Emulsifier OP-6 and CTAB. Based on the synthesis rate of the enantiomers calculated (Table 1), it was found that the rate of (*S*)-enantiomer (*v*_*S*_) increased while the rate of (*R*)-enantiomer (*v*_*R*_) decreased. In detail, the V_*S*_ increased from 0.072 to 0.97 mmol L^−1^ h^−1^ and 0.92 mmol L^−1^ h^−1^, as well as the V_*R*_ decreased from 0.009 to 0.002 mmol L^−1^ h^−1^ and 0.003 mmol L^−1^ h^−1^, respectively in the media with Emulsifier OP-6 and CTAB. The apparent reverse reaction rate (*v*_rev_) approached 0, which indicated no evidence of reverse reaction. In the system containing SDS, the phenomenon was quite different from the system containing Emulsifier OP-6 or CTAB.

**Table 1:** Effect of surfactants on reaction rates

Surfactant	*v*_0 *,R*_	*v*_0 *,S*_	*v*_*R*_	*v*_*S*_	*v*_rev_
Control	0.005	0.255	0.009	0.072	0.041
SDS	0.003	0.257	0.016	0.061	0.024
Emulsifier OP-6	0.003	0.347	0.002	0.097	0
CTAB	0.012	0.358	0.003	0.092	0

The unit was mmol L^−1^ h^−1^

### Effect of nonionic surfactants on the asymmetric reduction of 2-octanone

The Emulsifier OP series, including Emulsifier OP-6 (C_14_H_21_(OC_2_H_4_)_6_OH), Emulsifier OP-8 (C_14_H_21_(OC_2_H_4_)_8_OH), Emulsifier OP-10 (C_14_H_21_(OC_2_H_4_)_10_OH) and Emulsifier OP-12 (C_14_H_21_(OC_2_H_4_)_12_OH), contain an ethoxylate with the unit number ranging from 6 to 12. As seen in the Table 2, the length of hydrophilic ethoxylate had a marked influence on the reaction yield decreasing from 88.4 to 65.3% with the chain length.

**Table 2:** Relation between character of nonionic surfactants and cell catalytic activity

Surfactant	Hydrophobic end	Hydrophilic end	Yield (%)	Viability (%)	R_Glc_ (%)	OD_260 nm_	OD_280 nm_
Emulsifier OP-6	C_14_H_21_	(OC_2_H_4_)_6_OH	88.4	87.5	100	0.19	0.28
Emulsifier OP-8		(OC_2_H_4_)_8_OH	74.9	88.9	100	0.22	0.45
Emulsifier OP-10		(OC_2_H_4_)_10_OH	73.3	88.5	100	0.26	0.63
Emulsifier OP-12		(OC_2_H_4_)_12_OH	65.3	87.1	100	0.27	0.71
Tween 60	C_17_H_35_CO	(OC_2_H_4_)_5_OH × 3	56.6	62.7	100	0.12	0.27
Tween 80	C_17_H_33_CO		57.9	75.0	100	0.14	0.32
Tween 40	C_15_H_31_CO		60.6	67.5	100	0.17	0.38
Tween 20	C_11_H_23_CO		93.2	72.2	100	0.21	0.56

Cultural condition: 10 g L^−1^ glucose; 150 g L^−1^ wet cell; 0–2.0 mmol L^−1^ surfactants; 20 mL Tris–HCl buffer (50 mmol L^−1^, pH8.0); 32 °C; 200 r min^−1^; anaerobic; 24 h

Determination condition: 10 g L^−1^ glucose (10 mL); 100 g L^−1^ wet cell; aerobic; 32 °C; 200 r min^−1^; 4 h

Reaction condition: 0.2 g glucose; 150 g L^−1^ wet cell; 0.4 mmol L^−1^ surfactants; 20 mL Tris–HCl buffer (50 mmol L^−1^, pH 8.0); 32 °C; 200 r min^−1^; anaerobic; 24 h

**Fig. 2:**
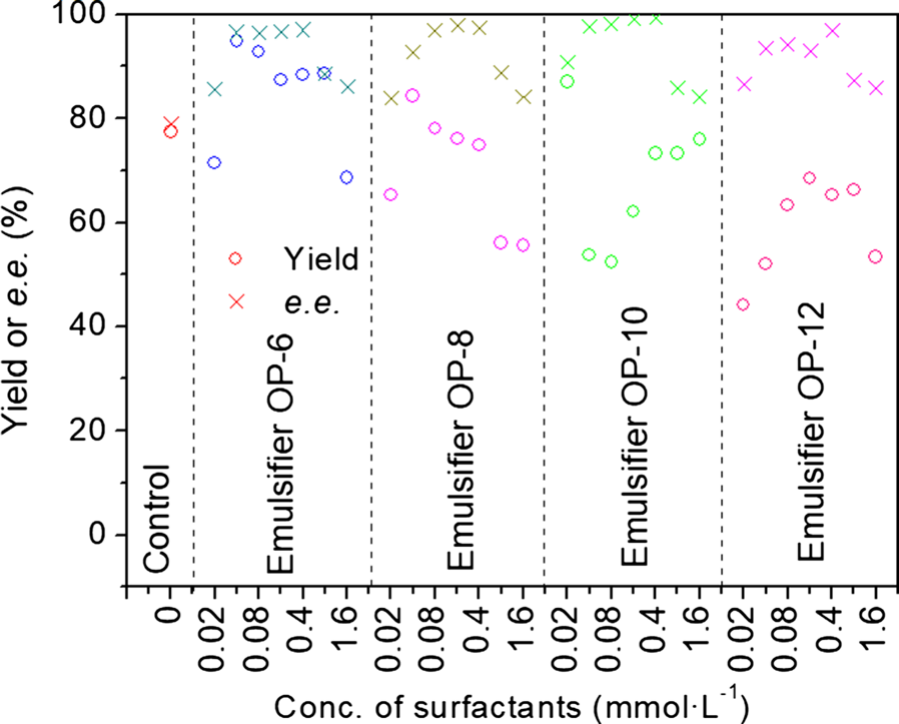
Effect of Emulsifier OP surfactants on asymmetric reduction of 2-octanone by baker’s yeast cells. Reaction condition: 10 mmol L^−1^ 2-octanone; 150 g L^−1^ wet cell; 10 g L^−1^ glucose/24 h; 20 mL Tris–HCl buffer (50 mmol L^−1^, pH 8.0); 32 °C; 200 r min^−1^; anaerobic; 96 h

The carbon chain length of ethoxylate in APEO series surfactants showed a mild influence on the product *e.e.* value. As shown in Fig. 2, the product *e.e.* values exhibited no distinct differences from each other in the reaction with diverse carbon chain length, excluding the difference due to the surfactant concentration. At the surfactant concentration of 0.4 mmol L^−1^, the product *e.e.* was maintained at a high value, for example, reaching 99.2% in the reaction with Emulsifier OP-10. However, the *e.e.* value decreased rapidly at a higher level of surfactant concentration.

Other ethoxylate surfactants, such as polyoxyethylene sorbitan fatty acid ester (namely Tween series surfactants), are obtained based on their degree of ethoxylate polymerization, for example, Tween 20 [polyoxyethylene (20) sorbitan monolaurate, CH_3_(CH_2_)_10_COO(OC_2_H_4_)_20_C_6_H_8_O(OH)_3_], Tween 40 [polyoxyethylene (20) sorbitan monopalmitate, CH_3_(CH_2_)_14_COO(OC_2_H_4_)_20_C_6_H_8_O(OH)_3_], Tween 60 [polyoxyethylene (20) sorbitan monostearate, CH_3_(CH_2_)_16_COO(OC_2_H_4_)_20_C_6_H_8_O(OH)_3_] and Tween 80 [polyoxyethylene (20) sorbitan monooleate, CH_3_(CH_2_)_7_CH=CH(CH_2_)_7_COO(OC_2_H_4_)_20_C_6_H_8_O(OH)_3_]. These compounds contain the same hydrophilic end but different hydrophobic terminal groups, like C_12_H_23_CO, C_16_H_31_CO, C_18_H_35_CO and C_18_H_33_CO (Table 2).

**Fig. 3:**
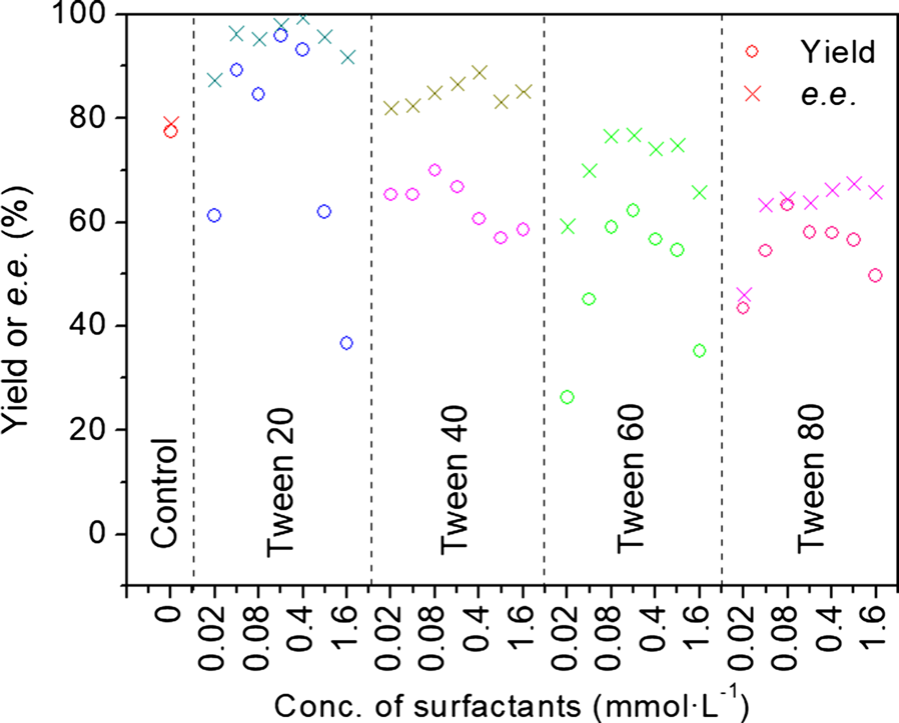
Effect of Tween surfactants on asymmetric reduction of 2-octanone with baker’s yeast cells. Reaction condition: 10 mmol L^−1^ 2-octanone; 150 g L^−1^ wet cell; 10 g L^−1^ glucose/24 h; 20 mL Tris–HCl buffer (50 mmol L^−1^, pH 8.0); 32 °C; 200 r min^−1^; anaerobic; 96 h

The concentration of the Tween series surfactants showed a significant effect on the *e.e.* value (Fig. 3). When the concentration of Tween 20 was 0.4 mmol L^−1^, the *e.e.* reached 99.3%. However, the *e.e.* dropped to about 92% with the increase of the concentration. Similarly, the concentration of the other three Tween series surfactants clearly affected the *e.e.* value.

The Tween series surfactants affected the yield in the *S. cerevisiae*-mediated reduction of 2-ocatanone depending on the length of hydrophobic end (Tween 60 > Tween 80 > Tween 40 > Tween 20). The shorter the carbon chain, the higher the yield (Fig. 3). The result was related to the biocompatibility of the hydrophobic end to the cell membrane. Increased biocompatibility associated with the longer hydrophobic end lowered the cell membrane permeability, and hence resulting in a lower yield (Table 2).

## Discussion

Surfactants are compounds with various molecular structures and charges (Gozde et al. 2017). Therefore, the impact of surfactants such as SDS, Emulsifier OP-6 or CTAB on living cells varies with the composition of the medium. Furthermore, these surfactants affect the reaction characteristics in the asymmetric reduction of 2-octanone with *S. cerevisiae* (Shi et al. 2010).

A rapid drop of (*S*)-enantiomer and the continuous increase of (*R*)-enantiomer of 2-octanol were observed after 48 h of the reaction. Data indicated two unexpected reactions: the oxidation of (*S*)-2-octanol to 2-octanone, and the side reaction of the reduction, namely the reduction of 2-octanone to (*R*)-2-octanol.

This phenomenon resulted from the excessive accumulation of (*S*)-enantiomer in the reaction process in the cell. The asymmetric reduction of ketone to alcohol is a reversible reaction, with the reaction rate and direction depending on the concentration of ketone, alcohol, coenzyme, and other factors. As the positive reaction rate decreased due to product inhibition, the relative concentration of byproduct (*R*)-2-octanol increased. At the same time, the reverse reaction occurred along with the decreased concentration of substrate 2-octanone in the reaction. These results suggested that the decrease of product concentration at the intracellular enzyme surface was the most important factor in improving the product yield and *e.e.* value (Shi et al. 2010).

The reverse and side-reactions involving the reduction of 2-octanone to (*S*)-2-octanol catalyzed by the whole cell *S. cerevisiae*, led to low yield and product *e.e.* value. Accordingly, the presence of Emulsifier OP-6 and CTAB, except SDS, not only improved the positive reaction rate, but also limited the reverse and side reaction rate effectively, which resulted in a better yield and product *e.e.* value.

The effects of APEO surfactants depend both on the hydrophobic alkyl and the length of ethoxylate. The effects due to the length of ethoxylate and alkyl are usually antagonistic (Christopher et al. 2018). A longer ethoxylate commonly leads to better solubility, but worse surfactant activity. The effect of Emulsifier OP series of surfactants on the asymmetric reduction of 2-octanone is worth further investigation.

The Emulsifier OP series, contain an ethoxylate with the unit number ranging from 6 to 12. The length of hydrophilic ethoxylate had a marked influence on the reaction yield decreasing with the chain length. Consequently, the weakened influence of the surfactants on the hydrophobic cell membrane is attributed to the higher hydrophilicity resulting from the increased length. Furthermore, the concentration of surfactants might be another important factor affecting the yield as the high concentration of surfactants is possibly associated with a decline in enzymatic activity, for example, plasma membrane (PM) vesicles isolated from the yeast *S. cerevisiae* (wild-type NCIM 3078, and a MG 21290 mutant pma 1-1) were used to monitor the effect of the nonionic surfactant Triton X-100, on (H^+^)-ATPase (E.C. 3.6.1.35), NADH oxidase and NADH-hexacyanoferrate (III)[HCF (III)] oxidoreductase (EC 1.6.99.3) activities. The results obtained, showed that Triton X-100 inhibited both membrane-bound and solubilized NADH-dependent redox activities (Awasthi et al. 2005). The nature of this inhibition as determined for NADH-HCF(III) oxidoreductase was non-competitive.

However, the carbon chain length of ethoxylate in APEO series surfactants showed a mild influence on the product *e.e.* value. The *e.e.* value decreased rapidly at a higher level of surfactant concentration. It confirmed that the properties of hydrophilic and hydrophobic groups, as well as the concentration of surfactants, but not the length of side chain of hydrophilic end, were the most important factors contributing to the product *e.e.* value.

Compared with Emulsifier OP series surfactants, the Tween series surfactants affected the yield in the *S. cerevisiae*-mediated reduction of 2-ocatanone depending on the length of hydrophobic end. The shorter the carbon chain, the higher the yield. Increased biocompatibility associated with the longer hydrophobic end lowered the cell membrane permeability, and hence resulting in a lower yield.

However, no correlation was observed between the cell membrane permeability caused by different series of surfactants and the reaction yield, as well as between the viability or R_Glc_ and the yield, based on the findings of Emulsifier OP series compounds (with fixed hydrophobic end and varied hydrophilic end) and Tween series compounds (with fixed hydrophilic end and varied hydrophobic end). These results illustrated that nonionic surfactants not only changed the cell membrane permeability (Liu et al. 2016) to alleviate the product inhibition, but also affected one or more of the intracellular enzymes (Kurakake et al. 2017), the cell status, and coenzyme regeneration. Further, these effects related with the class and length of both hydrophilic and hydrophobic ends of the compounds. Interestingly, the shorter length of both hydrophilic and hydrophobic ends improved the yield, irrespective of the surfactant type.

The carbon chain length at the hydrophobic and hydrophilic ends, and the concentration of surfactants were important factors affecting the product *e.e.* value (Figs. 2, 3 and Table 2). However, the carbon chain lengths of hydrophobic end had a more significant impact than the length of hydrophilic end. For example, no marked change in *e.e.* value resulted from the altered length of the hydrophilic end (Fig. 2), which was quite different from the degree of change caused by the length of hydrophobic end (Fig. 3). With C_11_H_23_CO (Tween 20) as the hydrophobic end, the average *e.e.* value reached 94.7%, which dramatically dropped to 49.5% when the hydrophobic end was C_17_H_33_CO (Tween 80).

The shorter carbon chain in the nonionic surfactants, improved the yield, irrespective of the length of hydrophobic or hydrophilic ends. The Tween series surfactants strongly affected the product *e.e.* value compared with the Emulsifier OP series surfactants. The carbon chain length of hydrophobic end had a significantly greater impact than the length of hydrophilic end in nonionic surfactants. The nonionic surfactants Emulsifier OP-10 and Tween 20 improved the yield and product *e.e.* value effectively. At a concentration of 0.4 mmol L^−1^, the reaction yield at 96 h was 73.3 and 93.2%, respectively. Furthermore, the *e.e.* value was 99.2 and 99.3%, which were significantly higher compared with the reaction without surfactants.

## Abbreviations

PEIC: phenylethyl isocyanate
OP: octylphenol polyoxyethylene
SDS: sodium dodecyl sulfate
CTAB: cetyltriethylammonium bromide
GC: gas chromatography
APEO: alkylphenol ethoxylates
